# An Event-Related Potential Study of the Neural Response to Inferred Motion in Visual Images of Varying Coherence

**DOI:** 10.3389/fpsyg.2019.02117

**Published:** 2019-09-13

**Authors:** Lei Jia, Yufan Xu, John A. Sweeney, Cheng Wang, Billy Sung, Jun Wang

**Affiliations:** ^1^Department of Psychology, Zhejiang Normal University, Jinhua, China; ^2^Department of Psychiatry and Behavioral Neuroscience, University of Cincinnati, Cincinnati, OH, United States; ^3^School of Marketing, Curtin University, Perth, WA, Australia

**Keywords:** extrastriate visual cortex, human medial temporal complex (hMT+), implied motion (IM), coherence levels, P2, event-related potential (ERP)

## Abstract

A vivid sense of motion can be inferred from static pictures of objects in motion. Like perception of real motion (RM), viewing photographs with implied motion (IM) can also activate the motion-sensitive visual cortex, including the middle temporal complex (hMT+) of the human extrastriate cortex. Moreover, extrastriate cortical activity also increases with motion coherence. Based on these previous findings, this study examined whether similar coherence level-dependent activity in motion-sensitive human extrastriate cortex is seen with IM stimuli of varying coherence. Photographic stimuli showing a human moving in four directions (left, right, toward, or away from the viewer) were presented to 15 participants. The coherence of the stimuli was manipulated by changing the percentage of pictures implying movement in one direction. Electroencephalographic data were collected while participants viewed IM or counterpart non-IM stimuli. The P2 response of extrastriate visual cortex (source located at hMT+) increased bilaterally with coherence level in the IM conditions but not in the non-IM conditions. This finding demonstrates that extrastriate visual cortical responses are progressively activated as motion coherence increases, even when motion is inferred, providing new support for the view that the activity of human motion-sensitive extrastriate visual cortex can be modulated by top-down perceptual influences in addition to its well-established role in processing bottom-up sensory signals.

## Introduction

The ability to perceive motion is a core feature of perceptual systems and has adaptive value in dynamic environments. Many studies have shown that the human motion-sensitive extrastriate visual cortex, which consists mainly of human middle temporal complex (hMT+/V5) and extrastriate body area (EBA), is the core cortical region supporting the processing of visual information about biological motion (Ungerleider and Desimone, 1986; Britten et al., 1992). Specifically, the hMT+, the homologue of the middle temporal (MT) and medial superior temporal (MST) areas in the macaque monkey brain, is sensitive to motion direction. In functional magnetic resonance imaging (fMRI) and magnetoencephalography (MEG) studies using random dot paradigms (RDPs), hMT+ responds more strongly to coherent motion than incoherent motion and its activity increases as motion coherence increases (Nakamura et al., 2003; Aspell et al., 2010). The EBA, which is found at the posterior end of the inferior temporal sulcus (partly overlapping hMT+) and is selectively activated by static images of human bodies and body parts, is also sensitive to visual information about biological motion (Peelen et al., 2006).

The sensitivity of extrastriate visual cortex (including hMT+ and EBA) is not limited to the processing of sensory motion signals (real motion, RM), but extends to implied motion (IM) detectable in static images. Using neuroimaging methods such as fMRI and MEG, previous studies have demonstrated that viewing static photographs which imply motion, such as the Enigma visual illusion, a cup falling off a shelf, or a photograph of an athlete running, can evoke higher hMT+ activity than viewing similar photographs without IM (Kourtzi and Kanwisher, 2000; Krekelberg et al., 2003; Kourtzi et al., 2008). In addition, there is also ERP evidence that observation of static pictures of dynamic body actions can increase activity of the hMT+ and EBA, which are part of human motion-sensitive extrastriate visual cortex (Proverbio et al., 2009). This evidence suggests that top-down processing of motion inferred from static images occurs in parallel with the bottom-up perception of real motion (RM). It is notable that IM responses in hMT+ are usually delayed compared with responses to RM (Lorteije et al., 2006; Fawcett et al., 2010).

The role of extrastriate visual cortex in perception of IM has, however, been challenged by new evidence from single-cell analyses in macaque TM/MST and fMRI of human hMT+ (Lorteije et al., 2011). Neither MT nor hMT+ discriminated IM figures from non-IM figures shown against a moving random dot pattern (RDP). Instead, activity was correlated with the low-level visual features of the moving random dots, such as orientation and size. Although these results might be confounded by factors such as attention and non-salient figures, there was no evidence of motion processing in hMT+ during IM and activity was related only to low-level visual features. On that basis, Lorteije et al. (2011) argued that IM processing in area MT/hMT+ could be better explained by sensitivity to low-level features than processing of IM.

**Figure 1:**
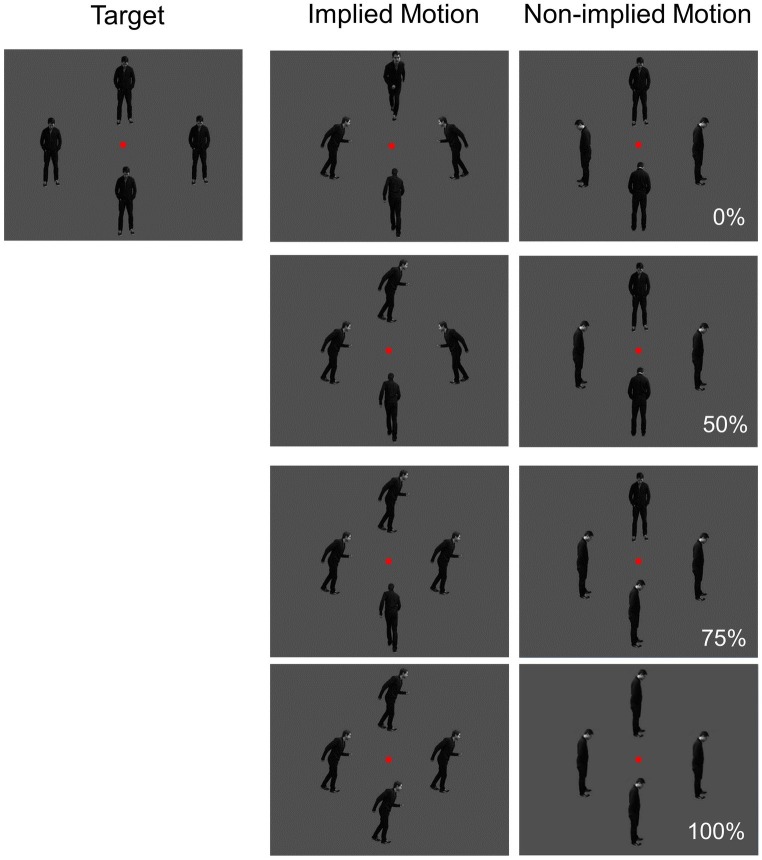
Visual stimuli used in the study. Directional coherence (0, 50, 75, and 100%) and implied motion (IM; non-IM) were manipulated.

In summary, there is conflicting evidence about whether the extrastriate visual cortex is involved in integration of low-level motion features or is a higher level, more specialist region involved in processing information about both IM and RM (Kourtzi et al., 2008; Lorteije et al., 2011). This of course raises questions about the role of top-down perceptual processes as well as bottom-up sensory processing of visual motion signals, and their potential integration, in the visual cortex. This study aimed to address this controversy using visual event-related potentials (VERPs, sometimes referred to as visual evoked potentials, VEPs). Previous MEG and EEG studies have shown that both early and later neural activity (<100 and >200 ms, respectively) are sensitive to IM. However, only the latter neural response to IM has been seen in hMT+ (Lorteije et al., 2006; Fawcett et al., 2010). Based on this knowledge, we examined extrastriate visual cortical responses to IM across images displaying varying levels of coherence. We used a simple task in which static photographs of humans running or standing still were employed as IM and non-IM stimuli respectively. Coherence (i.e., percentage of photographs of images moving in the same horizontal direction; see Figure 1) was manipulated by systematically changing the direction in which the human agent was running or facing. VEPs to IM and non-IM stimuli were recorded and analyzed.

## Materials and Methods

### Subjects

Fifteen healthy subjects (aged 19–34 years; 11 women) participated in the experiment. All participants were right-handed and had normal or corrected-to-normal vision. The Institutional Review Board of the University of Illinois at Chicago approved the study, and participants provided written informed consent (in accordance with the Declaration of Helsinki) prior to testing. Participants were paid $15/h for their participation.

### Materials and Procedures

Stimuli were presented on a 22-inch monitor at a viewing distance of 1 m. Three types of gray-scale pictures were used: implied motion (IM), non-implied motion (non-IM), and target (see Figure 1). IM stimuli consisted of three sets of four images of a single person surrounding a central fixation point at a distance of 2 degrees. In each image, the human agent was running left, right, toward, or away from the viewer. The non-IM stimuli also consisted of three similar sets of images; but in these images, the human agent was shown in a standing position, leaning left, right, toward, or away from the viewer. The target stimuli were sets of four images in which the human agent was standing facing toward the viewer.

Coherence was operationalized as the percentage of images facing toward or moving in a single horizontal direction; there were four levels (0, 50, 75, and 100%).

The trial procedure was similar to that used by Lorteije et al. (2006; Experiment 1). Each photograph in a set of four was presented for 500 ms, followed by a 1,000-ms inter-stimulus period during which a black screen was displayed. The order of presentation of the three trial types (IM, non-IM, and target) was randomized. Participants were instructed to fixate on the red dot at the center of the photographs and to press a button if the human was shown facing toward the viewer in all four images. Each participant completed 612 trials [276 IM trials (45.10%), 276 non-IM trials (45.10%), and 60 target trials (9.80%)]. Equal proportions of non-IM and IM images were used at each coherence level. The target trials were included to ensure that participants were attending to the stimuli.

### Recording and Analysis of EEG Data

EEG data were collected from 64 sintered Ag/AgCl sensors (Quik-Cap, Compumedics Neuroscan, Charlotte, NC) with a forehead ground and nose reference; impedance was kept below 5 kΩ. Electrodes placed at the outer canthi of both eyes and above and below the right eye were used to record vertical and horizontal eye movements. EEG data were digitized and recorded at 1,000 Hz continuously during testing.

Raw data were checked for bad channels (less than 5% for all participants), which were replaced using a spherical spline interpolation method (as implemented in BESA 5.1; MEGIS Software, Gräfelfing, Germany). Data were transformed to an average reference and digitally filtered from 1 to 40 Hz (12 db/octave roll-off, zero phase). Eye blink and cardiac artifact correction was carried out using the ICA toolbox in EEGLAB (Delorme and Makeig, 2004) implemented in Matlab software (MathWorks, Natick, MA).

**Figure 2:**
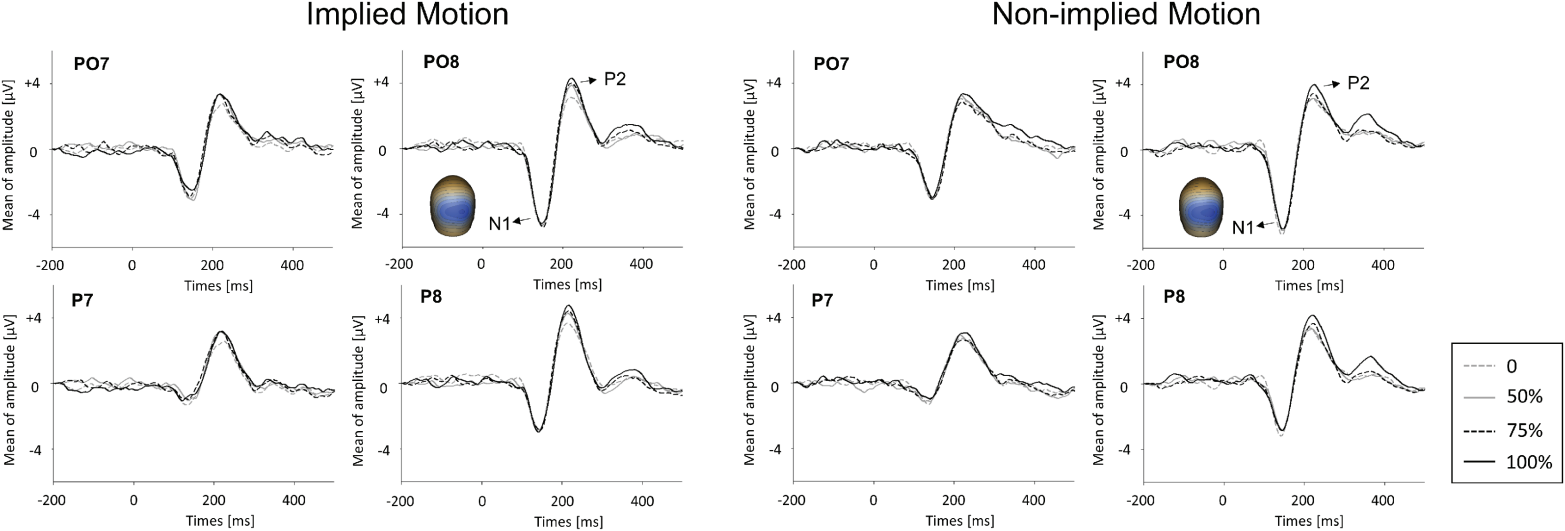
Event-related potential waveforms for implied motion (IM) pictures and non-implied motion (non-IM) pictures at different coherence levels. Two visual event-related potentials, N1 and P2, were identified. The global field power (GFP) plots are based on peak amplitudes. Averaged topographic maps of the N1 component (100–170 ms) in IM condition and non-IM condition are also shown.

Only VERPs elicited by non-target (IM and non-IM stimuli) trials to which no key press response was made (correct rejections) were included in the analyses. All participants generated more than 25 valid trials for each condition. Data from individual trials (200 ms before stimulus onset to 500 ms post-onset) were averaged separately for IM and non-IM stimuli at each coherence level. Trials with activity greater than 75 μV were automatically excluded from further processing. Grand averages were baseline-corrected using the 200-ms pre-stimulus period. Components above baseline noise level were identified by deriving global field power (GFP) plots for every subject and condition (Skrandies, 1989). The only identifiable components in the GFP plots of all subjects in all conditions were the N1 (100–170 ms) and P2 (170–260 ms; see Figure 2). The magnitudes of N1 and P2 (in μV) were quantified at the latency of the peak magnitude of the component (±4ms) by identifying the highest negative (N1) or positive (P2) reading from the voltage sensors over the posterior cortical region (commonly sensors around P7/P8 or PO7/PO8) and taking the average of this sensor and the four surrounding sensors (Wang et al., 2010, 2012).

A 2 (stimulus type: IM; non-IM) × 4 (coherence level: 0, 50, 75, and 100%) repeated-measures ANOVA was then conducted on GFP plots of VERP components (i.e., N1 and P2) to test for the potential effects on their amplitude. The Greenhouse–Geisser correction was applied to values of *p*.

### Localization of Visual Event-Related Potentials

After analyzing VERPs taken from voltage data at the sensors, we used L2 minimum norm (Hämäläinen and Ilmoniemi, 1984) available in BESA to assess the potential association between the extrastriate visual cortex and the VERPs observed in the sensor space data. In the final analyses, we used the largest magnitude source at each location.

## Results

### Event-Related Potential Results

#### N1 (100–170 ms)

**Figure 3:**
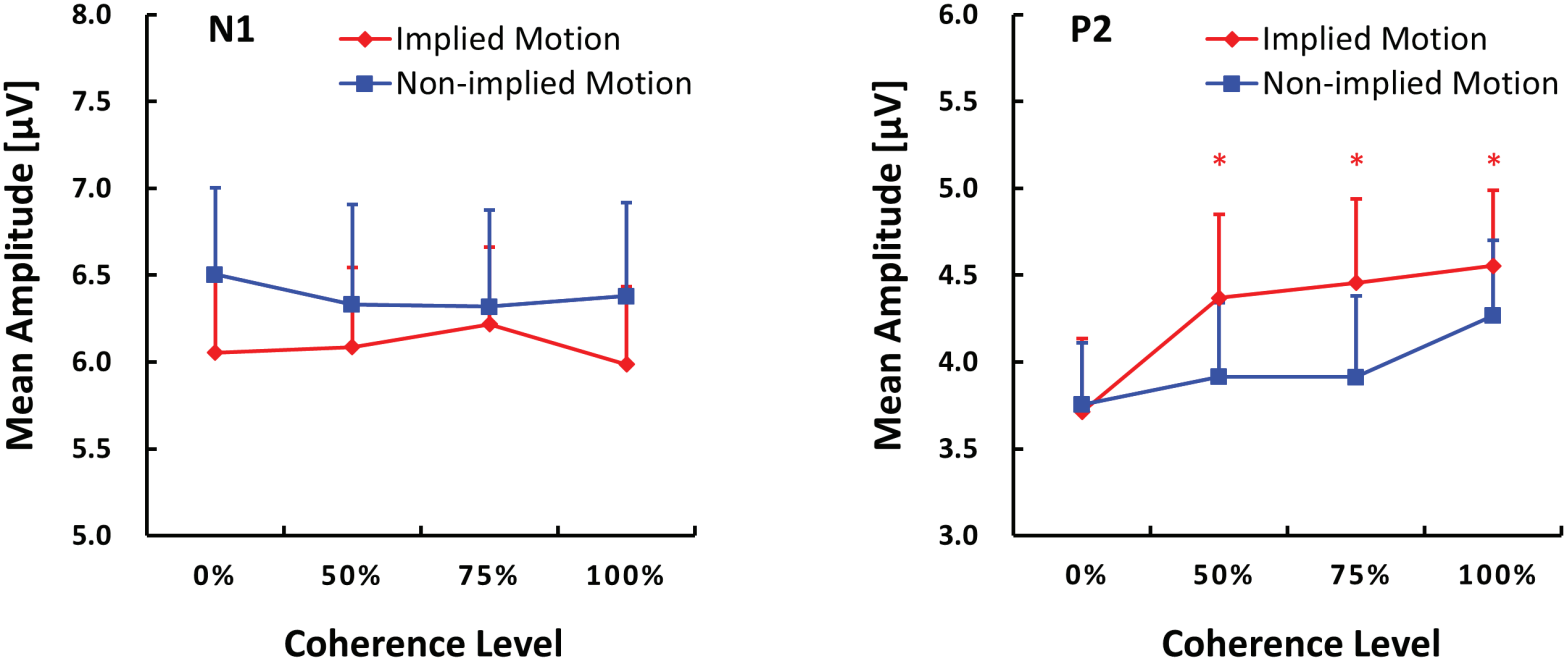
Global field power (GFP) plots of N1 and P2 components (M ± SE) evoked by implied motion (IM) pictures and non-implied motion (non-IM) pictures at different coherence levels. * indicates *p* < 0.05.

GFPs of VERP components are shown in Figure 3. ANOVA of N1 amplitude showed only a marginal effect of stimulus type (*F*_(1,14)_ = 3.55, *p* = 0.081, $\eta_{p}^{2}$ = 0.20). There was no main effect of coherence level (*F*_(3,12)_ = 0.20, *p* = 0.90) and no interaction between coherence and stimulus type (*F*_(3,12) =_ 0.33, *p* = 0.80).

#### P2 (170–260 ms)

ANOVA with P2 amplitude as the dependent variable revealed main effects of stimulus type (*F*_(1,14)_ = 5.96, *p* = 0.029, $\eta_{p}^{2}$ = 0.30) and coherence (*F*_(3,12)_ = 6.48, *p* = 0.007, $\eta_{p}^{2}$ = 0.62), as well as a stimulus type x coherence interaction (*F*_(3,12)_ = 4.35, *p* = 0.027, $\eta_{p}^{2}$ = 0.52). *Post hoc* tests of the effect of stimulus type revealed that IM photographs (*M* = 4.27, SE = 0.45) elicited a larger P2 response than non-IM photographs (*M* = 4.27, SE = 0.45). *Post hoc* tests of the coherence effect showed that P2 amplitude was lower at the 25% coherence level than that at the other coherence levels (*p* ≤ 0.018). No other pairwise differences between coherence levels were found.

For the stimulus types × coherence levels interaction on P2 amplitude, its simple effects can be seen in Figure 3. The amplitude of the P2 response was lower in IM trials than non-IM trials at coherence levels of 50, 75, and 100% (*p* ≤ 0.042); P2 amplitudes did not differ at the 0% coherence level. Furthermore, separate repeated-measures ANOVAs conducted for IM and non-IM trials revealed an effect of coherence on IM trials (*F*_(3,12)_ = 9.67, *p* = 0.002, $\eta_{p}^{2}$ = 0.71) but not non-IM trials (*F*_(3,12)_ = 2.67, *p* = 0.10). *Post hoc* tests of the coherence effect on IM revealed that P2 amplitude was lower at the 0% coherence level than at all other coherence levels, *p* ≤ 0.003.

### Source of Event-Related Potentials

**Figure 4:**
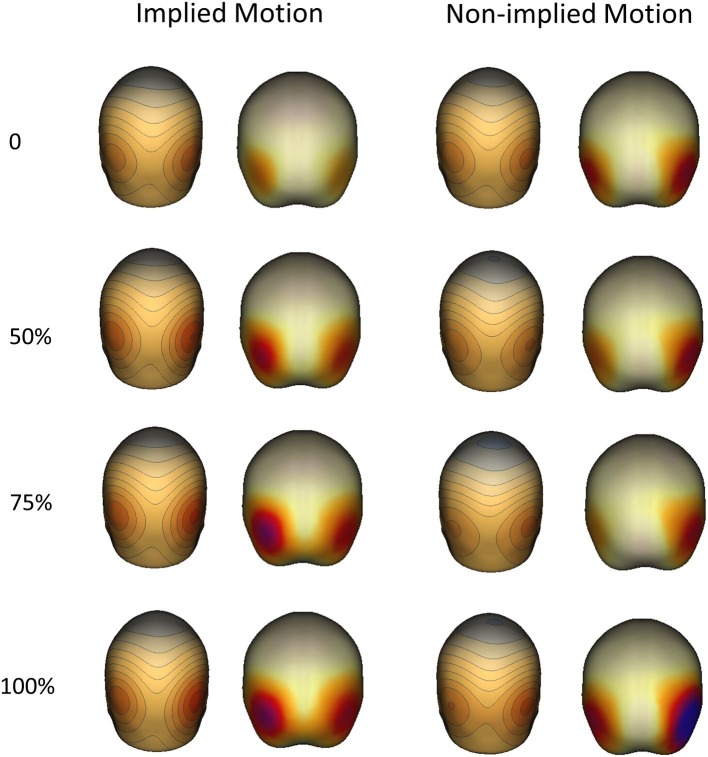
Topographical maps (right) and sources (left) of P2 components evoked by implied motion (IM) and non-implied motion (non-IM) images of humans at different coherence levels. Localization analyses indicated that bilateral hMT+ regions were associated with P2 activity.

Like previous EEG and MEG studies, we failed to find an association between the sources of the N1 (100–170 ms) responses and the location of hMT+ or other parts of the extrastriate visual cortex. However, the latter P2 response (170–260) was associated with bilateral posterior cortex activity in hMT+ regions, which was positively related to coherence in both IM and non-IM trials (see Figure 4).

## Discussion

Previous studies have produced inconsistent evidence on the role of hMT+ at the extrastriate visual cortex in RM and IM processing. It is well established that there is attentional modulation of MT activity, which implies that there is some top-down modulation of this brain region (e.g., Buchel et al., 1998), but whether hMT+ activity is modulated by top-down, higher order perceptual analysis of complex visual scenes remains controversial (Kourtzi et al., 2008; Lorteije et al., 2011). Hence this study examined activity in the extrastriate visual cortex (mainly hMT+) in response to photographs of humans showing IM at four coherence levels. We analyzed VERPs and found that P2 amplitudes, which were more robustly related to IM than earlier N1 responses in hMT+, varied systematically with coherence. Based on these results, the current study reveals two crucial findings on the roles of extrastriate visual cortex playing on motion selection.

This study adds to previous MEG and EEG studies on hMT+ of motion perception because we have demonstrated that the extrastriate visual cortex is sensitive to the coherence of IM, which adds to the well-established finding that this region is sensitive to the coherence of RM (Nakamura et al., 2003; Aspell et al., 2010). Our results indicate that the source of the P2 was in hMT+. However, due to the limited spatial resolution of EEGs and ERPs, we cannot exclude the possibility that the other body-selective regions [i.e., EBA, fusiform face area (FFA) and fusiform body (FBA)] which lie close to hMT+ were involved in this P2 neural network. Previous research has indicated that the latter body-selective regions are distinct from motion-selective hMT+, though they are also selectively activated by biological motion displays (Peelen et al., 2006; Proverbio et al., 2009). Nevertheless, our finding indicates that top-down perceptual processing of IM in static images can modulate processing of visual information in the extrastriate region dedicated to the processing of visual motion signals. This observation is consistent with previous studies indicating that RM and IM might share similar processing mechanisms. For example, they show similar time-dilation and motion-induced position shift (MIPS) effects (Yamamoto and Miura, 2012; Holmin et al., 2016).

In addition, our study also provides novel information about the role of motion-sensitive human extrastriate cortex in detection of IM and may thus extend knowledge of the top-down cortico-cortical influences that modulate the functioning of extrastriate visual cortex. Consistent with previous studies, our finding with regard to P2 amplitudes indicates that extrastriate visual cortex (particularly hMT+) is sensitive to motion (i.e., differentiates between images that imply motion and those that do not) and also processes of low-level visual feature (i.e., directional coherence). Moreover, the interaction of motion and coherence on P2 amplitude indicated that the coherence of IM images, but not non-IM images, modulated neural activity in the extrastriate visual cortex. In particular, in sets of images showing some directional coherence (i.e., 50, 75, or 100%), P2 amplitude was sensitive to motion, whereas in sets of images with no directional coherence, P2 amplitude was not modulated by IM. This finding indicates that, low-level visual features such as coherence may drive the motion detection system. Only the latter component of the neural response was sensitive to IM, which is consistent with a perceptual rather than sensory effect. A reasonable interpretation of this finding is that the motion selectivity of the extrastriate visual cortex is grounded in perceptual integration of low-level visual features (e.g., directions). Lack of coherent IM does less, perhaps because of direction selectivity but also possibly because of coherent percepts driving the system more dramatically. Furthermore, given the limited spatial resolution of EEG and ERPs, it is possible that hMT+ works together with other body-selective regions (i.e., EBA, FFA, and FBA) as part of a complex neural network that perceives motion in coherent percepts. Further work needs to be done to investigate this possibility empirically. Overall, our findings suggest a novel explanation for the unusual results of Lorteije et al. (2011).

## Data Availability

The raw data supporting the conclusions of this manuscript will be made available by the authors, without undue reservation, to any qualified researcher.

## Ethics Statement

The studies involving human participants were reviewed and approved by The Institutional Review Board of the University of Illinois at Chicago. The patients/participants provided their written informed consent to participate in this study.

## Author Contributions

LJ contributed to experiment design, data analysis, and draft writing. YX contributed to data analysis and draft writing. JS contributed to experiment data collection and drafting the article. CW contributed to data analysis. BS contributed to drafting the article. JW contributed to research conception, experiment design, and drafting the article.

### Conflict of Interest Statement

The authors declare that the research was conducted in the absence of any commercial or financial relationships that could be construed as a potential conflict of interest.
